# Combined larynx large cell neuroendocrine and squamous cell carcinoma: a case report

**DOI:** 10.31744/einstein_journal/2023RC0618

**Published:** 2023-11-23

**Authors:** Juliana Hesse, Leandro Aurélio Liporoni Martins, Leonardo Haddad, Fabio Pupo Ceccon

**Affiliations:** 1 Hospital Israelita Albert Einstein Faculdade Israelita de Ciências da Saúde Albert Einstein São Paulo SP Brazil Faculdade Israelita de Ciências da Saúde Albert Einstein, Hospital Israelita Albert Einstein, São Paulo, SP, Brazil.; 2 Hospital Israelita Albert Einstein São Paulo SP Brazil Hospital Israelita Albert Einstein, São Paulo, SP, Brazil.

**Keywords:** Laryngeal neoplasms, Carcinoma, neuroendocrine, Carcinoma, squamous cell, Head and neck neoplasms, Carcinoma, large cell

## Abstract

Laryngeal cancer ranks third among the most common head and neck neoplasms. The most common histological subtype is squamous cell carcinoma, and neuroendocrine tumors are rare. An even rarer entity is a composite tumor with both these histologies. This case reports a metastatic combined carcinoma of squamous cells and large neuroendocrine cells, presenting favorable response to treatment with a total laryngectomy followed by adjuvant therapy including chemo-, radio-, and immunotherapy.

## INTRODUCTION

Laryngeal cancer ranks third among the most common head and neck neoplasms and affects more men than women. Treatment options generally negatively affect the patient's quality of life. The primary risk factors for laryngeal cancer include alcohol and tobacco use, dietary habits, HPV infection, and occupational exposure.^(1)^ Staging follows the tumor–node–metastasis system. Treatment and prognosis differ considerably according to the exact anatomical structure involved as well as the tumor's histology. While the most common histological subtype is squamous cell carcinoma (SCC), neuroendocrine tumors are rare.^(2)^ Infrequently, they might appear simultaneously.

Herein, we describe a rare laryngeal combined squamous cell and large cell neuroendocrine carcinoma (LCNEC) and the course of treatment.

## CASE REPORT

**Figure 1:**
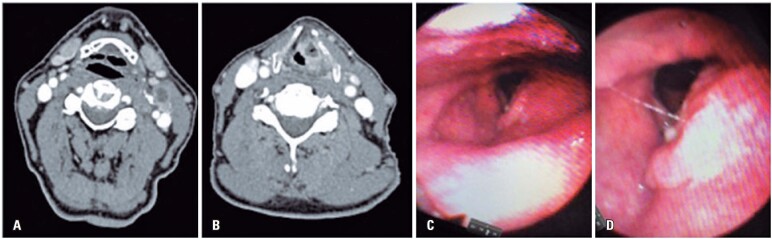
A) Axial section with level 2A metastasis on the left; B) Expansive, infiltrative, and ulcerated formation, with heterogeneous contrast enhancement, transglottic, affecting the glottic and supraglottic larynx on the left; C and D) Laryngoscopy images evidentiating the left vocal fold presenting vegetative and infiltrative lesion in the vestibular fold that reaches the petiole of the epiglottis. Fixation and immobility of the left vocal fold was observed. The right vocal fold had normal mobility and no mucosal lesion

A 64-year-old male smoker sought medical assistance due to 3-month long hoarseness. Physical examination revealed a lump of level 2 at the left side of his neck (Figures 1A and 1B); laryngoscopy showed a single mass fixing the left vocal cord extending to the vestibular fold with vegetative and infiltrative components (Figures 1C and 1D). Infraglottic lesions were not visualized.

Microlaryngoscopy was performed to biopsy the tumor, resulting in an invasive squamous cell carcinoma. After discussing the therapeutic options, the patient chose to undergo surgery and adjuvant postoperative chemo/radiotherapy.

Complete laryngectomy with left neck dissection from levels 1 to 6 was performed in the same month. Stapler closure of the pharynx and primary tracheoesophageal prosthesis insertion were done. The patient had a favorable recovery and was discharged on the 10^th^ day. One week later, he presented with a pharyngocutaneous fistula that was treated conservatively.

**Figure 2:**
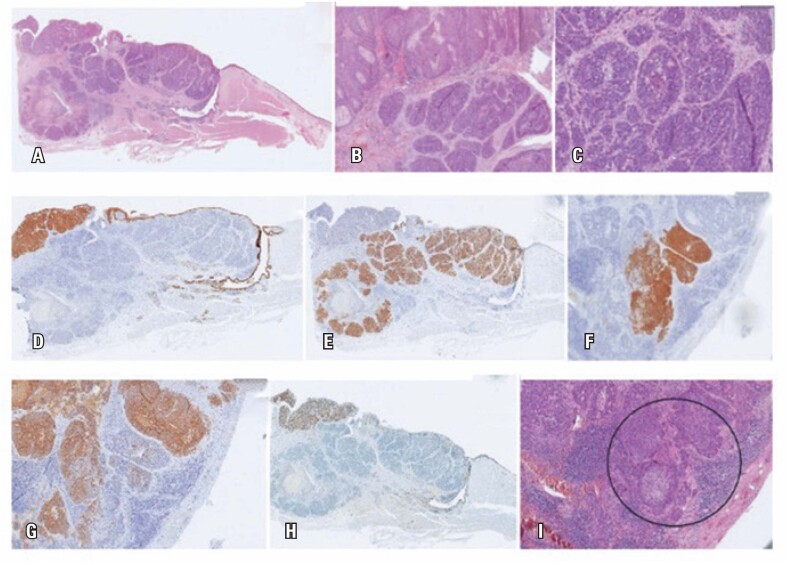
A) Hematoxylin and Eosin (HE) (larynx): longitudinal section showing the supraglottic neoplasm (the true vocal fold, on the right, is free from involvement). There is a superficial component on the upper left (in situ and invasive SCC) and a predominant component on the right and in the depth (LCNEC); B) Transition of SCC (upper half) and LCNEC (lower half) components; C) HE (larynx): detail of the predominant component (LCNEC), with a neoplastic block in the center showing rosettes and punctate necrosis; D) Immunohistochemistry (larynx): cytokeratin 5/6 confirming squamous differentiation from SCC; E) Immunohistochemistry (larynx): synaptophysin confirming neuroendocrine differentiation from the predominant component (neuroendocrine carcinoma); F) Immunohistochemistry (metastasis): cytokeratin 5/6 positive in the SCC component. Around, negative neoplastic blocks (component of neuroendocrine carcinoma); G) Immunohistochemistry (metastasis): positive synaptophysin in the neuroendocrine carcinoma component. In the center, negative neoplastic blocks (SCC component); H) Immunohistochemistry (larynx): p40 confirming squamous differentiation from SCC; I) HE (metastasis): two components present. In the circle, the squamous component, confirmed by immunohistochemical positivity of cytokeratin 5/6

The anatomopathological report demonstrated two primary tumors in the larynx: a predominant compound of grade 3 (high grade) LCNEC that displayed cellular anaplasia, evident nucleolus and high mitosis activity and another area of in situ and invasive SCC. These results allowed us to consider a combined carcinoma, with two components: squamous cell carcinoma and predominant large cell neuroendocrine carcinoma (Figure 2).

Neck dissection resulted in 2/35 positive lymph nodes; the largest one (0.9cm) evidenced only neuroendocrine carcinoma while the smaller one presented both histological types.

After discussion with our institution's tumor board, we decided to initiate treatment with Cisplatin and Etoposide induction chemotherapy; subsequently, radiotherapy was given for the next 3 months. Nine months post-surgery, an oncologic PET scan revealed pulmonary metastasis. Thereby, a combined scheme of Paclitaxel and Carboplatin in addition to immunotherapy with Pembrolizumab was prescribed.

The patient is being followed-up regularly for 2.5 years post-op, and presents excellent voice rehabilitation, no dysphagia, or neck complaints. His oncologist plans to continue immunotherapy along with Pembrolizumab, since the patient presented good response without image evidence of pulmonary metastasis until now.

The article was approved by the Research Ethics Committee of *Hospital Israelita Albert Einstein* (CAAE: 69311822.8.0000.0071; # 6.050.965).

## DISCUSSION

Large cell neuroendocrine carcinoma is a rare, poorly differentiated, and aggressive high-grade neoplasm, affecting mostly the larynx among head and neck tumors.^(3)^ It has a strong association with smoking.^(4)^ Patients usually develop distant metastatic disease within 2 years and present a reserved prognosis. The 5-year survival rate represents only 15% of the total cases reported.^(5)^ Large cell neuroendocrine carcinoma incidence was not yet properly explored due to its rareness and difficulty in establishing a histologic diagnosis and due to its recent reclassification as a new entity only in 2017 by the World Health Organization (WHO Classification of Head and Neck Tumor 2017). Yet, it comprises <10% of all laryngeal neoplasms, considering that SCC is the most common form of laryngeal cancer, and its incidence is estimated at 90%.^(6)^ Thus, a tumor simultaneously containing these two histological compositions becomes an even rarer entity.

To establish a diagnosis of LCNEC, some criteria need to be met. According to Strosberg et al.,^(4)^ it should be visible as organoid nesting, trabecular growth, rosettes, and peripheral palisading. The tumor cells should be medium-large sized with moderate to abundant cytoplasm. Other characteristic features include marked cellular pleomorphism, vesicular chromatin, and small to prominent nucleoli. Numerous mitoses and comedo necrosis are easily identified. They are positive for cytokeratins and at least one neuroendocrine marker (synaptophysin, chromogranin, or INSM1).

Overall, the principles of laryngeal cancer treatment are focused in promoting direct treatment for the cancer itself, laryngeal preservation, and voice rehabilitation.^(7)^ Treatment options should be extensively pondered between patient-physicians, particularly surgical procedures, due to its impact on the patient's quality of life. While SCC treatment options are thoroughly studied, laryngeal LCNEC still lack robust evidence for a standardized treatment. By virtue of that, treatment is often guided by LCNEC treatment assumptions from other sites, mainly the lungs.^(6)^ For laryngeal LCNEC, systemic treatment with chemo- and radiotherapy is advocated^(8)^ even in early disease stages to act against eventual micrometastasis.^(3)^ Cisplatin + Etoposide schemes as well as immunobiologic drugs seem to be effective,^(3,6)^ with emphasis on Pembrolizumab-a monoclonal antibody anti-PD1 that presents favorable outcomes in patients treating lung LCNEC.^(9)^ Managing this disease surgically still remains a challenge, and when a combined tumor is identified, as in this case, treatment should be guided by the histology of the most aggressive disease.^(8)^

Laryngeal SCC treatment is well established and guided according to the tumor's topography and staging. For stage III tumors, as in the reported case, treatment involves a shared decision-making between performing definitive radiotherapy or laryngectomy with neck dissection plus adjuvant chemotherapy and/or radiotherapy.^(7)^ The use of Pembrolizumab in head and neck SCCs has also been proven to be effective in metastatic neoplasms or disease recurrence after first-line chemotherapy, showing approximately 50% overall response rate and establishing good results in reducing tumor burden. Thus, Pembrolizumab administration could have been a key treatment in the case reported here, as it might act in both histological subtypes.

## CONCLUSION

The challenge of facing a composite tumor of this nature encompasses both diagnostic and treatment spheres. Despite the fact that the treatment performed showed a favorable response so far, the need for controlled trials to better determine a curative proposal is evident but distant, due to the rarity of the case.
